# Quantification of horizontal force for the EXER-GENIE® resisted sprint training device

**DOI:** 10.3389/fspor.2023.1231371

**Published:** 2023-08-30

**Authors:** Jamie J. Ghigiarelli, Keith J. Ferrara, Yang Yang, James D. Abrechsten, Veronica M. Barat, Katie M. Sell, Adam M. Gonzalez

**Affiliations:** ^1^ Department of Allied Health and Kinesiology, Hofstra University, Hempstead, NY, United States; ^2^ Department of Athletics, Adelphi University, Garden City, NY, United States

**Keywords:** load cell, winch, technology, pulling force, velocity

## Abstract

Sport performance coaches use a range of modalities to apply a horizontal force (*F_h_*) to athletes during resisted sprint training (RST). These modalities include parachutes, weighted vests, pulley devices, motored tethered devices, and, most notably, weighted sleds. Despite the widespread use of these devices, the resistance forces of the pulley devices have not been evaluated for reliability and accuracy. Therefore, the primary aim of this study is to quantify the *F_h_* of a commercially available pulley device (EXER-GENIE®) and determine how resistance force is related to the load settings on the device. The secondary aim is to identify the differences in the *F_h_* values between three EXER-GENIE® devices that use 36 m and 60 m ropes. The *F_h_* values in the Newtons (N) of the three EXER-GENIE® devices were analyzed using a motorized winch, a lead acid battery, and an S-beam load cell. Four 10 s winch-driven trials were performed using 15 different EXER-GENIE® loads, ranging from 0.028 kg to 3.628 kg, employing two different 36 m devices and one 60 m device. The mean ± standard deviation for *F_h_* was reported across the four trials for each load setting. All devices produced similar *F_h_* values across lighter load settings (loads ≤0.141 kg). However, at heavier loads (loads ≥0.226 kg), the 60 m device had *F_h_* values 50–85 N greater than those of the 36 m device. The coefficient of variation across the four trials was extremely high at light loads but sharply decreased to <10% at heavy loads. Absolute reliability was high for each device [intraclass correlation coefficient (ICC) = 0.99]. A regression analysis for *F_h_* values and EXER-GENIE® load indicated a strong positive relationship between load and *F_h_* values across all devices (*R*^2 ^= 0.96–0.99). Caution should be exercised when using identical loads on the different-length pulley devices, as the 60 m device produced greater *F_h_* values than the 36 m devices at load settings higher than 0.226 kg. These results can provide coaches and practitioners with a better understanding of the magnitude of resistance that is applied when prescribing EXER-GENIE® devices for higher training loads.

## 1. Introduction

A 2022 survey study found that 99% of strength and conditioning coaches prescribe speed development exercises (e.g., plyometrics, overspeed training, and resisted sprinting), and 63% of these coaches (in sports such as football, rugby, wrestling, and hockey) prescribe a method of resisted sprint training (RST) (1). The three methods of improving sprint speed and agility are categorized into primary (movement technique), secondary (resistance and assistance), or tertiary (flexibility and strength) (2). RST falls into the secondary category and involves using external loads to increase overall horizontal forces during the early acceleration phase of the sprint (i.e., 5–15 m) (3, 4). RST methods include weighted sleds (5–7), weighted vests, parachutes (8, 9), robotic tethered devices (e.g., 1080 Sprint™ and DynaSpeed™) (10–12), partner towing (13), and elastic resistance bands (14).

Weighted sled training is the most studied method of RST (4), and many strength and conditioning coaches have a favorable view of this method (15); however, there are several factors that must be considered when using weighted sleds as an RST tool. First, having multiple sleds can be a financial burden, as these devices are expensive. The cost of a 50 kg weighted sled can range from $300 to $400 (RogueFitness®, PerformBetter®). Second, having sufficient storage space for larger weighted sleds can be challenging.

Third, it is important to consider the surface where the weighted sled may be used. Different surfaces have different friction coefficients and, thus, provide different resistance levels for the same or similar sled weight. The resistive forces of the sled are highly dependent on the surface friction. The same sled may produce different levels of resistance, depending on weather conditions or the flooring surface (16, 17), which may lead to load variations between sessions or unknown levels of actual resistance. Finally, sled training can be time-consuming and laborious, as constant loading and unloading of weighted plates are required when training in a group setting or with multiple athletes simultaneously. While many professional sprint coaches have access to other training modalities, not all tools have been scientifically evaluated to quantify the force levels or evaluate training efficacy.

**Figure 1:**
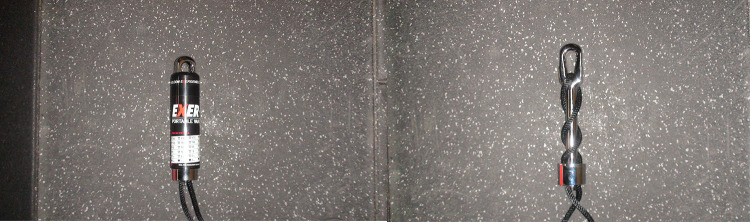
EXER-GENIE® device with and without the outer covering shell.

**Figure 2:**
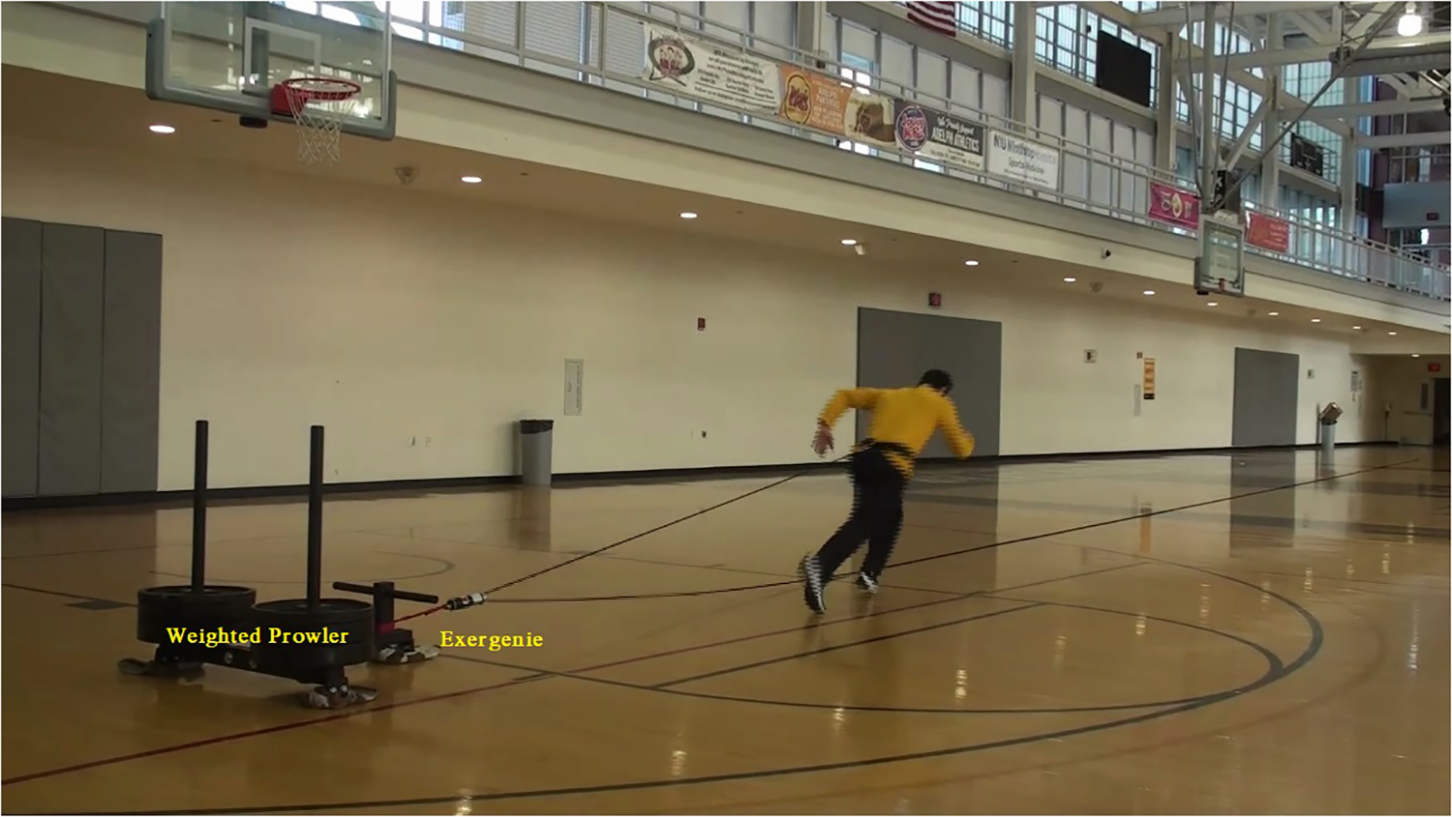
Use of the EXER-GENIE® device attached to a non-moveable loaded prowler.

The EXER-GENIE® (Thousand Oaks, CA) is a commercially available RST device (Figure 1). The unit weighs ∼14 oz and provides variable friction via a pulley system made of woven nylon rope that generates resistance by wrapping around an aluminum, nickel-plated, and chrome-coated shaft inside a heat-resistant, protective cylinder. The rope enters through an eyelet at the top of the metal shaft, progressively wraps around the shaft, and exits through a hole at the base of the device. When the bottom part of the EXER-GENIE® turns clockwise, the rope twists around the shaft, escalating frictional force, which progressively increases the resistance when pulling the rope. Conversely, twisting the device counterclockwise decreases the resistance. The device must be anchored by a non-moveable object and attached to a harness or belt on the athlete. Once the resistance is set, the athlete sprints in the direction against the resisted load (Figure 2 and Supplementary Video S1).

To date, there is no peer-reviewed research on the EXER-GENIE®, with the exception of several outdated dissertations (18–20) and unpublished data from the National Aeronautics and Space Administration (NASA) (21), which found a significant disparity between the actual force produced and the load printed on the device. This disparity reached as high as an 11-fold difference between the actual and the recorded values, and some EXER-GENIE® devices showed varying degrees of inaccuracy between repetitions. The researchers concluded that the EXER-GENIE® provided a wide range of resistive loads and was versatile, allowing astronauts in a hypo-gravity environment to complete a range of exercises. However, the inconsistency in load accuracy, as well as concerns related to excessive heat generation, were severe limitations of the device.

Although there is a lack of formal research on the EXER-GENIE®, anecdotal evidence shows that its use is prevalent among sprint coaches (22). The portability and light weight of the EXER-GENIE® allow for easy setup and transport, and resistance can be changed progressively and quickly. The device can also produce resistance in any direction, which optimizes coordination and neuromuscular performance in several planes of motion (23) or allows for adaptability to the physical layout of the training facilities. In addition, several EXER-GENIE® devices can be set up at the same time from the same anchor point, allowing greater efficiency for group testing without the manual labor of loading and de-loading weighted plates.

Given the potential benefits and unknown scientific accuracy of the EXER-GENIE®, this study aims to quantify the horizontal force (*F_h_*) data from the EXER-GENIE® across a uniform velocity using a load cell and motorized winch system. Computing *F_h_* using indirect methods, such as those used in the present study, is of considerable interest because the data can be easily integrated into the field to monitor training and loading, particularly for resisted sprinting (17). The secondary aim of this study is to report the differences between three EXER-GENIE® devices, namely, two 36 m devices and one 60 m device. Given our experiences in this field, we hypothesize that there would be discrepancies between the 36 m and the 60 m devices, in that the 60 m device would produce a greater *F_h_* than the 36 m one, despite identical load settings. The findings of this study will provide coaches and practitioners with a better understanding of the EXER-GENIE® load and the disparities between devices as well as the differences across devices when using the EXER-GENIE® in their respective programs.

## 2. Materials and methods

### 2.1. Experimental design

The *F_h_* of three EXER-GENIE® devices, namely, two separate 36 m devices (A and B) and one 60 m device (C), was analyzed using a motorized winch system, a lead acid battery, and an S-beam load cell. A similar methodology has been used to measure the dynamic frictional force when pulling a weighted sled (24, 25). The EXER-GENIE® has 48 load settings. The first 15 load settings range from 0.028 kg to 3.628 kg and were tested as part of this study, as these were primarily used as part of our off-season RST program. Four winch trials were performed at each load, producing 60 trials per device and a total of 180 trials across all devices.

### 2.2. Equipment

**Figure 3:**
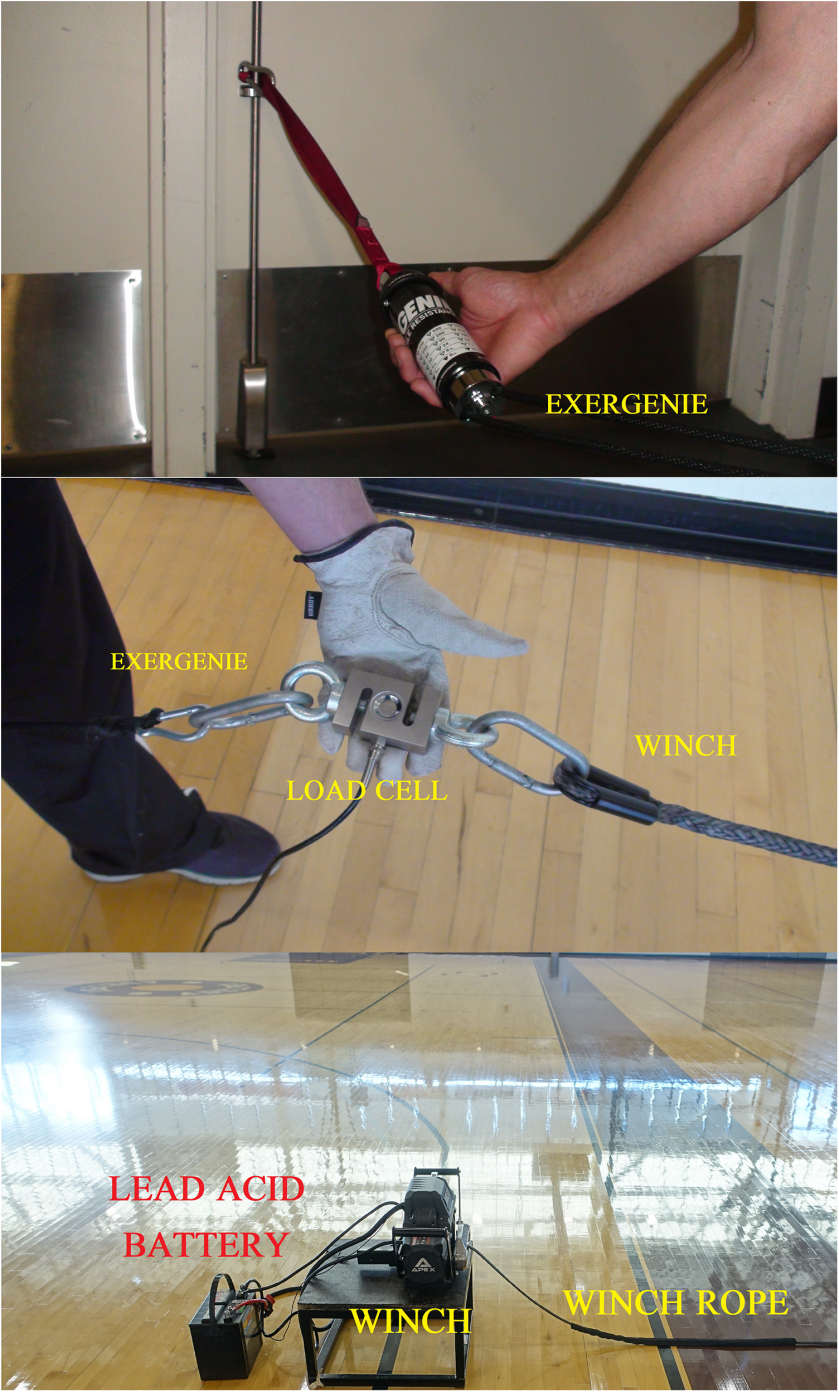
Components of the force-testing experimental trials, consisting of the winch, lead acid battery, load cell, and EXER-GENIE®.

A 24DC lead acid EverStart battery (Johnson Controls, Cork, Ireland) powered a non-movable motorized winch (Badland Apex, Model 56385, motor 12 VDC series wound, Calabasas, CA) (Figure 3). After pilot testing and experimental trial sessions, each battery was charged to full power using a 12 V-2/8/15 Amp Viking battery charger (Calabasas, CA). An S-beam load cell, with a maximum load capacity of ±500 kg (MuscleLab, Ergotest Innovation, Norway), was attached between the EXER-GENIE® (anchored to a double doorway) and the winch. The load cell had a 14-bit resolution with a sampling rate of 200 Hz. The temperature of the EXER-GENIE® was recorded by using an Ames Instruments™ Infrared Thermometer (AMES Instruments™, Calabasas, CA). The instrument is mechanized via a trigger that uses a laser and infrared lens to record the emissivity of the surface of an object, leading to a temperature reading. Preliminary work in our lab found that the internal temperatures of the EXER-GENIE® device reached 65° Celsius after 1 hour of use.

## 3. Procedures

### 3.1. EXER-GENIE® force testing

The winch was used to pull the EXER-GENIE® at a constant velocity across the normal friction force loads labeled on the device. The pulling velocity of the winch was 0.16 m·s^−1^, which is similar to the velocities used to analyze *F_h_* by Cross and colleagues (25). Notably, Cross et al. had a more robust winch system that was able to assess *F_h_* using a weighted sled on a track using a range of velocities (0.1–6 m·s^−1^). At the start of each trial, the length of the EXER-GENIE® and winch tether was 16 m and 8 m, respectively.

**Figure 4:**
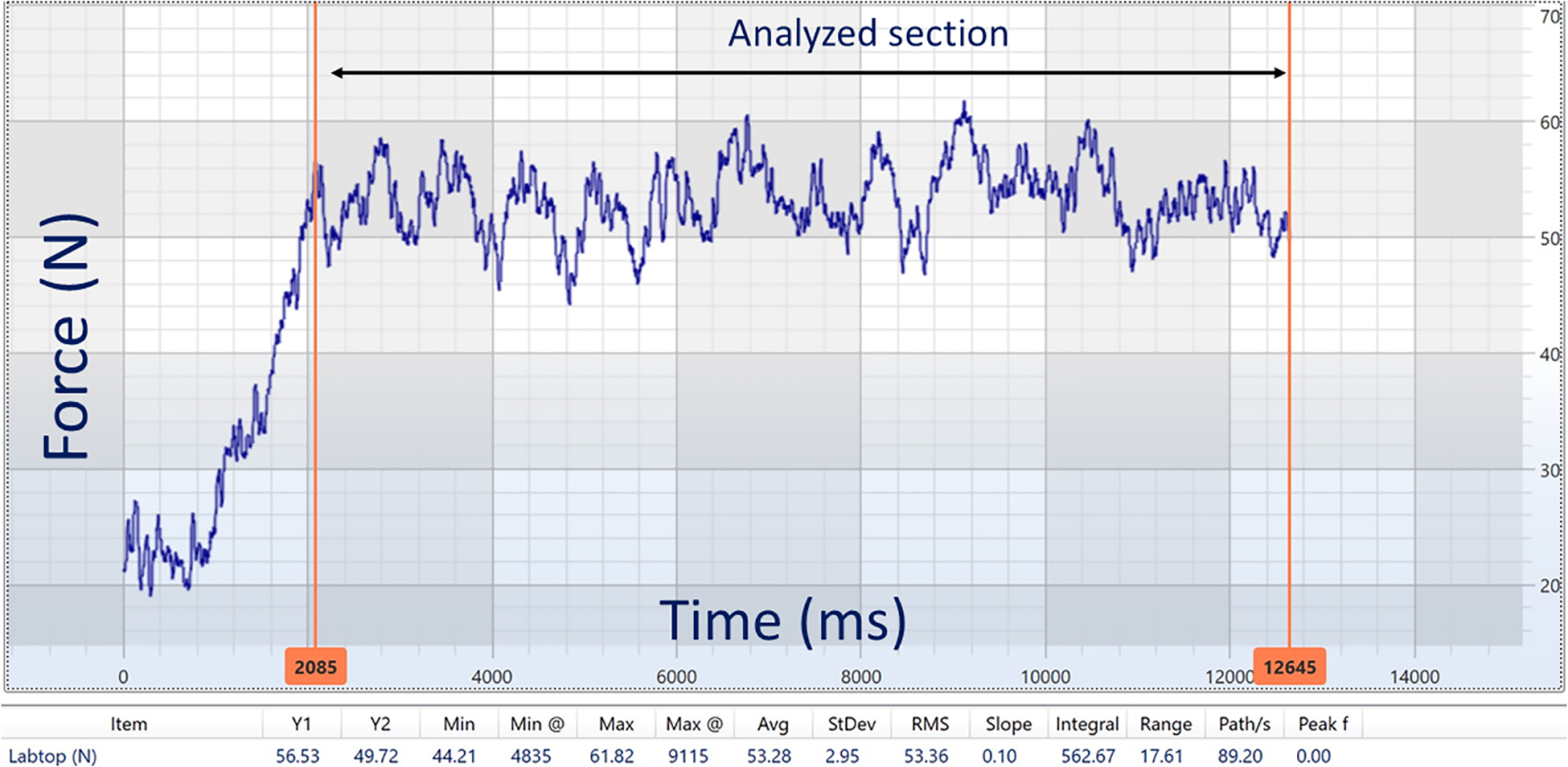
Raw force–time data and a selected analyzed section from a typical winch trial.

The load cell was zeroed twice for calibration before each trial. Initially, the load cell was placed horizontally on the ground. A subsequent calibration was performed after the primary investigator lifted the load cell off the ground and set it even with the height of the winch rope and EXER-GENIE® at 40.5 cm (see Supplemental Video S2). Aligning the height of the load cell, the EXER-GENIE®, and the winch rope eliminated non-zero angles of pull in the vertical direction. This methodology is consistent with that of previous research (25) and must be considered when calculating *F_h_* (17). The raw force–time data for a typical trial were collected for 10 s post-calibration, and the average force data within the time window were recorded. Figure 4 displays the raw force–time data and a selected analyzed section from a typical winch trial. For each trial, the data were analyzed after the initial spike, as force remained constant throughout the pull. Each trial was collected using MuscleLab Windows software Professional Edition and exported to a Microsoft Excel spreadsheet for further analysis.

### 3.2. Statistical analysis

Descriptive data (mean ± standard deviation) were calculated using IBM SPSS, Version 28.0 (SPSS Inc., Chicago, IL), software for determining the average *F_h_* for each EXER-GENIE® device across the four trials at the 15 loads. The coefficient of variation [CV = 100×(SD/mean)] was calculated for each trial. The mean CV% across the four trials with 95% confidence intervals (CI) was reported. Absolute reliability was calculated by using an intraclass correlation coefficient (ICC) with 95% CI using a two-way mixed model, with absolute agreement between the loads across the trials for each device. Fitted linear regression was used (GraphPad Prism version 9.2 for Windows; GraphPad software, La Jolla, CA) to examine the regression analysis between the *F_h_* and the EXER-GENIE® load displayed on the devices.

## 4. Results

### 4.1. Horizontal force data

Table 1 reports the descriptive and reliability *F_h_* data for each device across the 15 load settings. All three devices produced similar *F_h_* values across lighter load settings (loads ≤0.141 kg). As the loading progressively increased (loads ≥0.226 kg), devices A and B had similar *F_h_* values, while device C had higher *F_h_* values, with differences that ranged from 50 N to 85 N. The CV% was extremely high at light loads for each device; however, the CV% sharply decreased to below 10% at loads greater than 0.453 kg. Absolute reliability across the loads was high for each device. The average measures ICC and 95% CI for devices A, B, and C were 0.999 (0.998–0.999), 0.997 (0.994–0.999), and 0.998 (0.995–0.999), respectively.

**Table 1:** Mean ± standard deviation of tensile force in Newtons (N) and absolute reliability statistics within and between the loads in kilograms (kg) for the resisted sprint training devices A, B, and C at 0.16 m·s^−1^.

Load (kg)	Device A 36 m (N)	CV%	ICC	Device B 36 m (N)	CV%	ICC	Device C 60 m (N)	CV%	ICC
0.028	1.20 ± 2.06	205.4 (−23.2–434.1)	0.999 (0.998–0.999)	2.22 ± 2.14	125.5 (2.7–248.2)	0.997 (0.994–0.999)	1.65 ± 2.61	162.9 (21.8–304.0)	0.998 (0.995–0.999)
0.056	2.94 ± 1.70	58.6 (42.4–74.8)		3.67 ± 2.13	68.1 (5.3–131)		2.62 ± 2.39	94.5 (41.4–147.6)	
0.085	3.48 ± 2.19	86.3 (6.3–166.3)		4.11 ± 2.39	69.5 (−0.43–139.4)		3.99 ± 2.25	70.3 (5.5–135.1)	
0.141	3.63 ± 2.35	72.5 (−13.3–158.4)		4.28 ± 2.51	66.3 (22.5–110.2)		4.69 ± 2.39	69.2 (−17.0–155.4)	
0.226	4.83 ± 2.44	52.1 (33.6–70.5)		7.36 ± 2.61	41.1 (5.8–76.3)		12.30 ± 2.87	23.4 (17.6–29.3)	
0.283	5.50 ± 2.69	61.6 (3.9–119.3)		10.46 ± 2.30	23.8 (12.2–35.4)		20.78 ± 3.24	15.6 (13.6–17.6)	
0.340	8.12 ± 2.98	36.7 (32.5–41.0)		15.05 ± 2.89	19.9 (12.1–27.7)		26.75 ± 3.14	11.8 (9.7–13.9)	
0.453	18.24 ± 2.62	8.6 (7.2–10.0)		22.52 ± 2.81	12.6 (10.2–15.1)		49.42 ± 3.71	7.6 (5.5–9.7)	
0.907	30.57 ± 3.25	10.8 (8.3–13.3)		32.63 ± 3.33	10.2 (7.6–12.8)		80.88 ± 4.39	5.4 (4.1–6.7)	
1.360	48.81 ± 3.09	6.4 (4.4–8.3)		53.93 ± 3.99	7.4 (4.8–10)		102.70 ± 5.98	5.8 (4.6–6.9)	
1.814	69.81 ± 3.83	5.5 (4.6–6.4)		77.45 ± 6.53	8.2 (3.8–12.6)		142.42 ± 9.31	6.5 (3.5–9.6)	
2.267	111.16 ± 5.81	5.2 (2.7–7.7)		117.81 ± 7.87	6.7 (5.3–8.1)		170.51 ± 11.62	6.8 (2.5–11.1)	
2.721	139.45 ± 7.87	5.6 (4.2–7.0)		138.01 ± 11.55	8.3 (5.5–11.1)		204.18 ± 12.99	6.2 (2.6–9.8)	
3.175	168.33 ± 8.92	5.3 (3.5–7.1)		168.10 ± 16.98	10.0 (6.7–13.3)		255.90 ± 11.19	4.3 (2.3–6.4)	
3.628	228.57 ± 10.76	4.7 (3.9–5.5)		237.52 ± 10.38	4.3 (3.7–5.2)		279.34 ± 18.88	6.8 (3.5–10.0)	

ICC, intraclass correlation coefficient at 95% confidence interval; CV%, coefficient of variation at 95% confidence interval.

**Figure 5:**
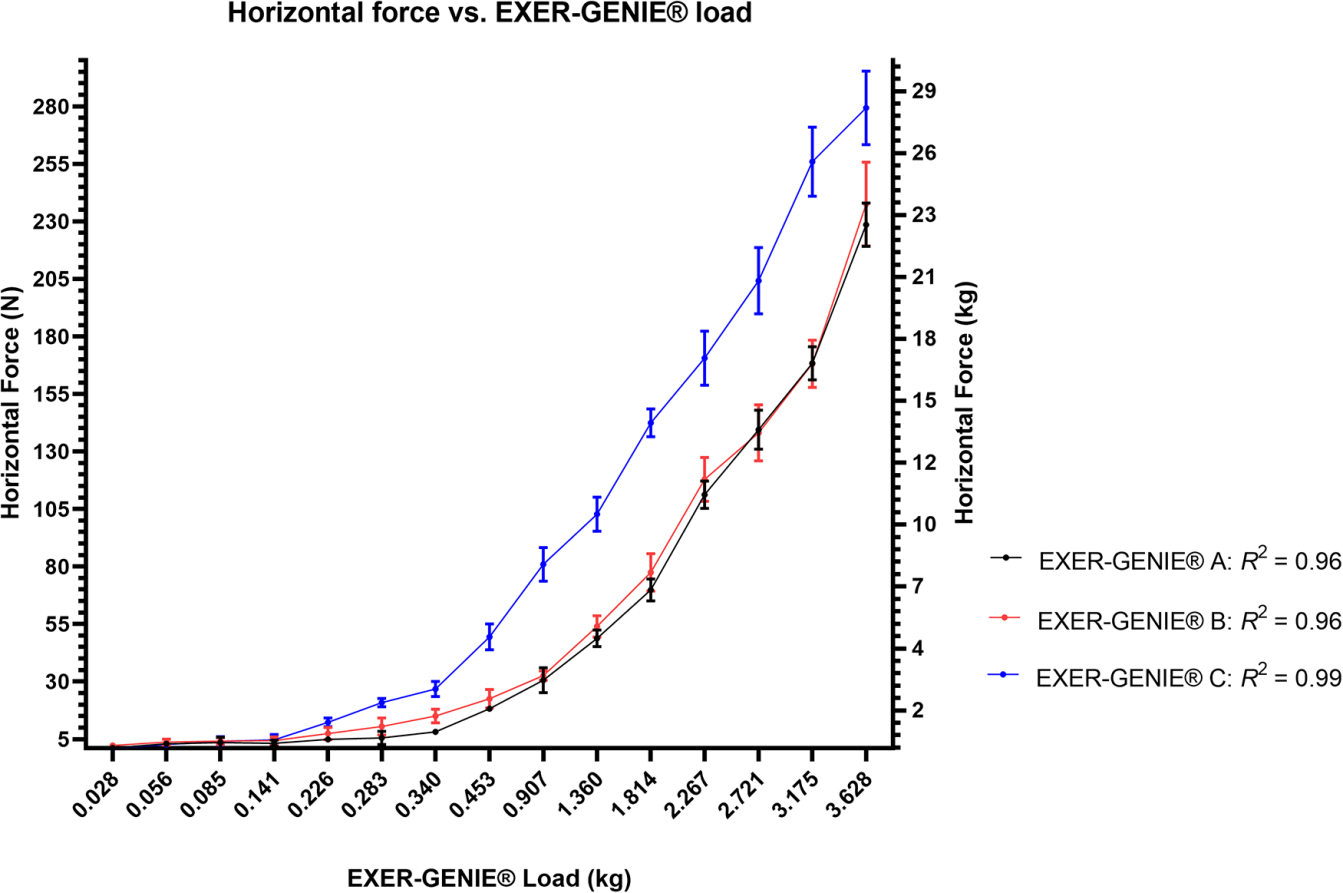
Horizontal force in Newtons (N) and kilograms (kg) vs. EXER-GENIE® load (kg) linear regression analysis across all three devices: (**A,B)** (36 m) and (**C**) (60 m).

There was a strong positive linear relationship between the *F_h_* values and the displayed EXER-GENIE® load (Figure 5). For device A (*R*^2^ = 0.966, *p* < 0.001), the regression equation was *F_h_* = 56.93 × (EXER-GENIE® load) − 9.95, and the 95% CI for the slope and the intercept were 50.56–63.61 and −20.65–0.73, respectively. For device B (*R*^2^ = 0.966, *p* < 0.001), the regression equation was *F_h_* = 57.54 × (EXER-GENIE® load) − 7.29, and the 95% CI for the slope and the intercept were 51.06–64.02 and −18.17–3.57, respectively. For device C (*R*^2^ = 0.996, *p* < 0.001), the regression equation was *F_h_* = 77.58 × (EXER-GENIE® load) + 0.24, and the 95% CI for the slope and the intercept were 74.61–80.54 and −4.73–5.23, respectively. In addition to the general linear regression analysis, we calculated two polynomial regression equations for the 36 m devices, as a visual inspection displayed a non-linear trend at very high EXER-GENIE® loads. To support this trend, the loading on the device progresses in non-linear increments beyond 3.628 kg. For device A, the polynomial equation was *F_h_* = 11.451*x*^2 ^+ 19.432*x* + 1.41, *R*^2 ^= 0.996. For device B, the polynomial equation was *F_h_* = 10.971*x*^2 ^+ 21.614*x* + 3.59, *R*^2 ^= 0.993.

## 5. Discussion

When testing up to a 3.628 kg setting, the EXER-GENIE® device produced *F_h_* values that ranged from 225 N to 280 N for the 36 m and 60 m devices. The value of 280 N is similar to that for other RST devices such as the 1080 Sprint™ and Dynaspeed™, which, according to manufacturer specifications, have a maximum loading of 300 N (30 kg) and 500 N (50 kg), respectively. A 2022 validity study using the 1080 Sprint™ used loads up to 110 N, which is equivalent to 2.267 kg on the 36 m EXER-GENIE® (12). There was a clear linear relationship between the load on the device and the *F_h_* value on the rope during the pulling trials. These *F_h_* values were similar between the two 36 m devices; however, *F_h_* values were higher for the 60 m device at higher loads. This finding supports our hypothesis and is consistent with what is reported during training programs.

The rationale for testing two separate 36 m devices was to examine the differences in manufacturing consistency. Ostensibly, both devices would produce the same force outputs because they would have similar constructs. Our data support this assumption, even though device B produced slightly larger *F_h_* values for about half the EXER-GENIE® loads compared with device A. It is important to note the difference in *F_h_* data obtained from the 60 m and the 36 m devices, despite identical load settings. The *F_h_* values were 50 N–85 N higher in the 60 m device, which may have important consequences when providing an athlete with consistent or ramped training loads. This was experienced in our pilot tests and is likely attributed to the length and weight of the rope; an extra 24 m may add considerable resistive force during the sprinting activity. Considering that this is a pulley system, when pulling the nylon rope in one direction, the remaining rope must be pulled in the opposite direction, which increases the overall inertia, mass, and resistance during the pull.

The CV% value range of 4–200% for the lighter loads is not surprising. The nylon rope lacks stability at light loads (<0.283 kg) due to minimal tension on the rope. When using heavier EXER-GENIE® loads, the rope increased in tension, resulting in more stability and producing less variation in *F_h_* values. Understanding the relationship between the *F_h_* value and the EXER-GENIE® loads across all three devices (Figure 5) can help determine the appropriate EXER-GENIE® load for a resisted sprint program design.

Loads beyond 3.628 kg were not tested in this study. A review by Zabaloy et al. (4) indicated that applying too high of a load during RST became counterproductive, as the athlete exhibited detrimental changes in sprint technique, such as “marching,” instead of normal propulsive sprinting mechanics. However, it is unclear whether the long-term effects of heavy RST training on unresisted sprinting techniques are negative. Lahti et al. (26) reported that heavy RST training at a 60% velocity decrement (*V*_dec_) for 9 weeks resulted in no adverse changes in the unresisted sprint technique. Further, the literature supports the benefits of short-term (4 weeks) and long-term (8–10 weeks) heavy RST training (i.e., *V*_dec_ 50%–80%, sled load of ∼90% body mass) on horizontal force production in the early acceleration phase and power output (27–30). Given the linear relationship between the tested loads and the resistive force in this study, the regression equations can be used to calculate a higher *F_h_* value that may occur during extremely heavy training events, such as the “truck pull,” which is commonly seen among strongman competitors (31).

This study has certain limitations. First, studies that measure *F_h_* values are inconsistent across the literature and can be complex; therefore, caution should be exercised in interpreting the data (17). Our methods required that the rope be pulled at a single constant velocity (0.16 m·s^−1^), which was not as robust as the motorized winch system in Cross, Tinwala (25), which pulled at a range of velocities (i.e., 0.1–6 m·s^−1^). According to the work–kinetic energy theorem, $FD=1/2mv^{2}$ (32), a faster constant velocity will increase kinetic energy, thus producing a higher *F_h_* output. Therefore, higher force outputs would be observed with greater pulling velocities, similar to what is seen in sprinting. Our motorized winch system, however, was more advanced than other studies, such as those of Andre et al. (24), which involved a mechanical winch, and Linthorne and Cooper (16), which involved pulling a weighted sled by hand. Further, Linthorne and Cooper (16) measured *F_h_* using a spring balance and measured constant velocity through timing gates.

Second, the use of *F_h_* as a primary outcome should also be considered when applying these findings to the clinical use of the device. Although *F_h_* provides extremely important information about the horizontal resistive forces, other variables such as *V*_dec_ may also play an important role when selecting loading parameters (27–29).

Third, the load settings of the EXER-GENIE® are prescribed in discrete quantitative amounts, which is different from the case with weighted sleds. The EXER-GENIE® provides 48 specific loads, whereas sleds can be loaded with any weight amount. From a training perspective, this could be problematic when determining optimal loads using *V*_dec_ because the prescribed load might fall between fixed intervals.

A fourth limitation is the angle of pull. For achieving greater accuracy in quantifying the *F_h_* value, the rope in this study was placed in the horizontal direction to minimize an angle of pull, thus eliminating a vertical component that could not be calculated by our load cell. In sprinting, however, an angle of pull off of the horizontal component would likely exist due to the varying heights of the athletes and the anchor points of the EXER-GENIE® (see Figure 2). The location of where the EXER-GENIE® rope attaches to the athlete and where the EXER-GENIE® is anchored may not be the same, thus affecting resistive force. Fortunately, the versatility of the EXER-GENIE® allows for attaching to a higher anchoring point, which could mitigate this problem.

Future research could quantify the *F_h_* value of the EXER-GENIE® for a hot device. Although anecdotal, users and coaches have reported a possible shift toward lower resistance at the same load setting for a hot device compared with a cold one. Future studies should also quantify the *F_h_* value for higher settings, as research has shown that loads greater than 28 kg, which is the highest kilogram load for this study (device C, 60 m), have been used successfully with weighted sleds (33).

In conclusion, the results from this study provide coaches and practitioners with a better understanding of the *F_h_* value produced by the EXER-GENIE® devices. The device produces horizontal forces greater than 220 N (∼22.5 kg) when testing up to 3.628 kg. The 36 m devices produce similar *F_h_* values across loads of 0.028–3.628 kg for a constant velocity. Finally, beyond 0.226 kg, the 60 m device produces greater *F_h_* values than the 36 m devices at the same load setting. Coaches must account for this difference when using the EXER-GENIE® device for sprint training and program design.

## Data Availability

The raw data supporting the conclusions of this article will be made available by the authors without undue reservation.

## References

[1] Weldon A Duncan MJ Turner A LaPlaca D Sampaio J Christie CJ. Practices of strength and conditioning coaches: a snapshot from different sports, countries, and expertise levels. J Strength Cond Res. (2022) 36:1335–44. 10.1519/JSC.0000000000003773 33298715

[2] Nicholson B Dinsdale A Jones B Till K. The training of short distance sprint performance in football code athletes: a systematic review and meta-analysis. Sports Med. (2021) 51:1179–207. 10.1007/s40279-020-01372-y 33245512 PMC8124057

[3] Rumpf MC Lockie RG Cronin JB Jalilvand F. Effect of different sprint training methods on sprint performance over various distances: a brief review. J Strength Cond Res. (2016) 30:1767–85. 10.1519/JSC.0000000000001245 26492101

[4] Zabaloy S Freitas TT Pareja-Blanco F Alcaraz PE Loturco I. Narrative review on the use of sled training to improve sprint performance in team sport athletes. Strength Cond J. (2023) 45:13–28. 10.1519/SSC.0000000000000730

[5] Zisi M Stavridis I Agilara GO Economou T Paradisis G. The acute effects of heavy sled towing on acceleration performance and sprint mechanical and kinematic characteristics. Sports (Basel). (2022) 10:1–10. 10.3390/sports10050077 PMC9146810 35622486

[6] Pareja-Blanco F Pereira LA Reis VP Fernandes V Arruda AFS Guerriero A Impact of sled loads on performance and kinematics of elite sprinters and rugby players. Int J Sports Physiol Perform. (2022) 17:465–73. 10.1123/ijspp.2020-0867 34965512

[7] Fernández-Galván LM Casado A García-Ramos A Haff GG. Effects of vest and sled resisted sprint training on sprint performance in young soccer players: a systematic review and meta-analysis. J Strength Cond Res. (2022) 36:2023–34. 10.1519/jsc.0000000000004255 35510888

[8] Martínez-Serrano A Marín-Cascales E Spyrou K Freitas TT Alcaraz PE. Electromyography, stiffness and kinematics of resisted sprint training in the specialized SKILLRUN® treadmill using different load conditions in rugby players. Sensors (Basel). (2021) 21:1–12. 10.3390/s21227482 PMC8622140 34833557

[9] Gleadhill S Kai T Nagahara R. Resist-and-release sprint running using parachute towing causes detrimental changes to performance, kinematics, and kinetics. J Phys Ed Sport. (2020) 20:3411–9. 10.7752/jpes.2020.06461

[10] Carlos-Vivas J Perez-Gomez J Eriksrud O Freitas TT Marín-Cascales E Alcaraz PE. Vertical versus horizontal resisted sprint training applied to young soccer players: effects on physical performance. Int J Sports Physiol Perform. (2020) 15:748–58. 10.1123/ijspp.2019-0355 32000140

[11] van den Tillaar R. Effect of active resisted 30 m sprints upon step and joint kinematics and muscle activity in experienced male and female sprinters. J Sports Sci. (2021) 39:1060–9. 10.80/02640414.2020.1856463 33258414

[12] Rakovic E Paulsen G Helland C Haugen T Eriksrud O. Validity and reliability of a motorized sprint resistance device. J Strength Cond Res. (2022) 36:2335–8. 10.1519/jsc.0000000000003830 35916750

[13] Chaalali A Bouriel K Rouissi M Chtara M Mkaouer B Cronin J Resisted sprint training with partner towing improves explosive force and sprint performance in young soccer players—a pilot study. Biol Sport. (2022) 39:379–87. 10.5114/biolsport.2022.103574 35309532 PMC8919874

[14] Le Scouarnec J Samozino P Andrieu B Thubin T Morin JB Favier FB. Effects of repeated sprint training with progressive elastic resistance on sprint performance and anterior-posterior force production in elite young soccer players. J Strength Cond Res. (2022) 36:1675–81. 10.519/jsc.0000000000004242 35622112

[15] Williams J Baghurst T Cahill MJ. Current perceptions of strength and conditioning coaches use of sled tow training. Int J Sports Sci Coach. (2021) 16:601–7. 10.1177/1747954120988618

[16] Linthorne NP Cooper JE. Effect of the coefficient of friction of a running surface on sprint time in a sled-towing exercise. Sports Biomech. (2013) 12:175–85. 10.1080/14763141.2012.726638 23898689

[17] Cross MR Tinwala F Lenetsky S Brown SR Brughelli M Morin JB Assessing horizontal force production in resisted sprinting: computation and practical interpretation. Int J Sports Physiol Perform. (2019) 14:689–93. 10.1123/ijspp.2018-0578 30975007

[18] Ainsworth JL. Effect of isometric-resistive exercises with the EXER-GENIE on strength and speed in swimming. Fayetteville, AR: Dissertation University of Arkansas (1970).

[19] O’Quinn MVD. Comparison of conventional and in-plane exer-genie training techniques in developing a forward passer. Fayetteville, AR: Dissertation Univesity of Arkansas (1968).

[20] Waddle B. Study comparing the effectiveness of a training program utilizing the EXER-GENIE with two conventional training programs on the development of muscular strength and cardiovascular endurance. Tallahassee, FL: Dissertation Florida State University (1967).

[21] Schaffner G Sharp C Stroud L. EXER-GENIE® exercise device hardware evaluation. Houston, TX: NASA Johnson Space Center. (2008). p. 1–18 Available from: https://ntrs.nasa.gov/citations/20080047032 (Accessed August 24, 2013).

[22] Valle CF. How to maximize the EXER-GENIE® in sports training. Simplifaster. (2019). Available from: https://simplifaster.com/articles/exer-genie-sports-training/ (Cited Jan 4, 2023).

[23] McBurnie AJ Parr J Kelly DM Dos’Santos T. Multidirectional speed in youth soccer players: programming considerations and practical applications. Strength Cond J. (2022) 44:10–32. 10.1519/SSC.0000000000000658

[24] Andre MJ Fry AC Bradford LA Buhr KW. Determination of friction and pulling forces during a weighted sled pull. J Strength Cond Res. (2013) 27:1175–8. 10.1519/JSC.0b013e318269aaef 22964856

[25] Cross MR Tinwala F Lenetsky S Samozino P Brughelli M Morin J-B. Determining friction and effective loading for sled sprinting. J Sports Sci. (2017) 35:2198–203. 10.1080/02640414.2016.1261178 27905864

[26] Lahti J Huuhka T Romero V Bezodis I Morin JB Häkkinen K. Changes in sprint performance and sagittal plane kinematics after heavy resisted sprint training in professional soccer players. PeerJ. (2020) 8:e10507. 10.7717/peerj.10507 33362970 PMC7747683

[27] Lahti J Jiménez-Reyes P Cross MR Samozino P Chassaing P Simond-Cote B Individual sprint force–velocity profile adaptations to in-season assisted and resisted velocity-based training in professional rugby. Sports (Basel). (2020) 8. 10.3390/sports8050074 PMC7281595 32466235

[28] Morin JB Capelo-Ramirez F Rodriguez-Pérez MA Cross MR Jimenez-Reyes P. Individual adaptation kinetics following heavy resisted sprint training. J Strength Cond Res. (2022) 36:1158–61. 10.519/jsc.0000000000003546 32058358

[29] Cahill MJ Oliver JL Cronin JB Clark K Cross MR Lloyd RS Influence of resisted sled-pull training on the sprint force–velocity profile of male high-school athletes. J Strength Cond Res. (2020) 34:2751–9. 10.1519/jsc.0000000000003770 32773545

[30] Derakhti M Bremec D Kambič T Ten Siethoff L Psilander N. Four weeks of power optimized sprint training improves sprint performance in adolescent soccer players. Int J Sports Physiol Perform. (2022) 17:1343–51. 10.123/ijspp.2020-0959 34706340

[31] Winwood PW Keogh JW Harris NK. The strength and conditioning practices of strongman competitors. J Strength Cond Res. (2011) 25:3118–28. 10.1519/jsc.0b013e318212daea 21993033

[32] Hamill J Knutzen K. Biomechanical basis of human movement. Philadelphia, PA: Wolters Kluwer Health/Lippincott Williams and Wilkins (2015). 372–85.

[33] Cahill MJ Oliver JL Cronin JB Clark KP Cross MR Lloyd RS. Sled-pull load-velocity profiling and implications for sprint training prescription in young male athletes. Sports (Basel). (2019) 7. 10.3390/sports7050119 31137511 PMC6572326

